# Modulation of chemically induced renal carcinogenesis by Chrysin via inhibition of oxidative stress, hyper-proliferation and inflammation at preclinical stage

**DOI:** 10.1186/2051-1426-3-S2-P69

**Published:** 2015-11-04

**Authors:** Summya Rashid, Sarwat Sultana

**Affiliations:** 1 Department of Medical Elementology and Toxicology, Jamia Hamdard (Hamdard University), New Delhi, India

## 

The present study was planned to investigate the chemopreventive efficacy of Chrysin (CH) against renal carcinogenesis in Wistar rats. Ferric nitrilotriacetate (Fe-NTA) is a potent nephrotoxicant and known renal carcinogen. CH is a natural flavonoid found in honey, propolis, blue passion flower. In vitro data suggests that it has anti-oxidative, anti-inflammatory, anti-apoptotic properties. Renal carcinogenesis is a multistep process and it originates from a series of molecular and histopathological alterations. Renal cancer was initiated by single intraperitoneal injection of N-nitrosodiethylamine and promoted chronically by twice weekly administration of Fe-NTA for 16 weeks and rats were sacrificed after 24 weeks. CH was administered at two doses daily. The possible mechanism could be induction of oxidative stress via upregulation of Lipid peroxidation (LPO), protein carbonyl (PC) and xanthine oxidase (XO); cellular proliferation via up regulation of Proliferative cell nuclear antigen (PCNA), Ki-67 and ornithine decarboxylase (ODC) along with reduction of inflammation by down regulating nuclear factor kappa B (NFkB), Cyclooxygenase-2 (Cox-2), inducible nitric oxide (iNOS), tumor necrosis factor-α (TNF-α), interleukin-6 (IL-6) and prostaglandin E2 (PGE2). Prophylactic treatment of CH mitigated serum toxicity markers, oxidative stress markers, reduced tumor incidences, down regulated proliferation and inflammation. These results provide a powerful evidence for the chemo preventive efficacy of CH at pre-clinical stage.

